# Divergent Metabolic Changes in Rhizomes of Lowland and Upland Switchgrass (*Panicum virgatum*) from Early Season through Dormancy Onset

**DOI:** 10.3390/plants12081732

**Published:** 2023-04-21

**Authors:** Nathan A. Palmer, Gautam Sarath, Michael J. Bowman, Aaron J. Saathoff, Serge J. Edmé, Robert B. Mitchell, Christian M. Tobias, Soundararajan Madhavan, Erin D. Scully, Scott E. Sattler

**Affiliations:** 1 Wheat, Sorghum, and Forage Research Unit, Agricultural Research Service, United States Department of Agriculture, Department of Agronomy and Horticulture, University of Nebraska-Lincoln, Lincoln, NE 68583, USA; nathan.palmer@usda.gov (N.A.P.); ajsaathoff1@gmail.com (A.J.S.); serge.edme@usda.gov (S.J.E.); rob.mitchell@usda.gov (R.B.M.); scott.sattler@usda.gov (S.E.S.); 2 Bioenergy Research Unit, National Center for Agricultural Utilization Research, Agricultural Research Service, United States Department of Agriculture, 1815 North University St., Peoria, IL 61604, USA; michael.bowman@usda.gov; 3 Division of Plant Systems-Production, National Institute of Food and Agriculture, United States Department of Agriculture, Beacon Complex, Kansas City, MO 64133, USA; christian.tobias@usda.gov; 4 Department of Biochemistry, University of Nebraska-Lincoln, Lincoln, NE 68588, USA; msoundararajan1@unl.edu; 5 Stored Products Insect and Engineering Research Unit, Agricultural Research Service, United States Department of Agriculture, Manhattan, KS 66502, USA; erin.scully@usda.gov

**Keywords:** abscisic acid, dormancy, metabolites, raffinose-family oligosaccharides (RFO), rhizomes, RNA-Seq, switchgrass (*Panicum virgatum* L.), transcriptomics

## Abstract

High-biomass-yielding southerly adapted switchgrasses (*Panicum virgatum* L.) frequently suffer from unpredictable winter hardiness at more northerly sites arising from damage to rhizomes that prevent effective spring regrowth. Previously, changes occurring over the growing season in rhizomes sampled from a cold-adapted tetraploid upland cultivar, Summer, demonstrated a role for abscisic acid (ABA), starch accumulation, and transcriptional reprogramming as drivers of dormancy onset and potential keys to rhizome health during winter dormancy. Here, rhizome metabolism of a high-yielding southerly adapted tetraploid switchgrass cultivar, Kanlow—which is a significant source of genetics for yield improvement—was studied over a growing season at a northern site. Metabolite levels and transcript abundances were combined to develop physiological profiles accompanying greening through the onset of dormancy in Kanlow rhizomes. Next, comparisons of the data to rhizome metabolism occurring in the adapted upland cultivar Summer were performed. These data revealed both similarities as well as numerous differences in rhizome metabolism that were indicative of physiological adaptations unique to each cultivar. Similarities included elevated ABA levels and accumulation of starch in rhizomes during dormancy onset. Notable differences were observed in the accumulation of specific metabolites, the expression of genes encoding transcription factors, and several enzymes linked to primary metabolism.

## 1. Introduction

Switchgrass (*Panicum virgatum* L.) is a temperate, warm-season, perennial grass with good attributes as a forage, conservation, and bioenergy crop [1,2]. Switchgrass occurs as populations and synthetic cultivars that have distinct zones of adaptation [3,4]. The more northerly adapted upland types possess greater winter hardiness, but have lower biomass yields compared to the higher-yielding lowland southerly lines which have lower winter survival in more northern climates. Although switchgrass accessions can be grown in different ecoregions [5], the average expected deployment is generally one hardiness zone north or south of the place of origin of a specific switchgrass accession [6]. However, some lowland switchgrass accessions appear to possess a few genotypes that can overwinter successfully at northern latitudes [7], suggesting that different winter/dormancy adaptive mechanisms could exist within the diverse switchgrass germplasm [8].

Growth of switchgrass starts in late April or early May depending on climatic conditions, followed by rapid vegetative growth in June, then transition to flowering and seed formation in July to August. Once seeds are mature, the aerial parts of the plant begin to senesce by late August/September, a period when the onset to dormancy occurs in below-ground tissue. In adapted germplasm, dormancy is established prior to a killing frost (normally November in Nebraska, USA). Adaptations for dormancy and onset to dormancy are accompanied by significant changes in tissue metabolism, changes in hormone levels, reordering of the transcriptome, and cessation of growth [9,10,11]. Prior to dormancy, there is normally an accumulation of storage reserves—mostly as starch—elevated levels of osmoprotectants, and a shift in metabolism from one of active growth to one of tissue maintenance. Delayed changes in any of these processes can lead to winter-kill [12]. A lack of winter survival in perenniating tissues of switchgrass leads to stand losses and ultimately to loss of sustainably producing biomass. Improving yields and winter hardiness have been important components of several US switchgrass breeding programs [1,7]. Extending the climatic range for high-yielding switchgrass lines without sacrificing winter hardiness and related biomass production advantages would be beneficial for crop deployment.

Palmer et al. [11] developed the first comprehensive atlas of the transcriptional and metabolic changes occurring over the course of two growing seasons in rhizomes sampled from field-grown upland Summer plants. Several key features of rhizome metabolism, especially during the onset of dormancy, were found. These findings included a pivotal role for ABA during dormancy, the increase in free sucrose in dormant rhizomes, and a redirection in rhizome metabolism to use stored reserves (starch, other polymers), more efficient recycling of C and N, and apparent greater reliance on substrate-level generation of ATP and reducing equivalents. Other data documented the transcriptional and metabolite profiles linked to rhizome growth processes [11].

The major focus of this study was to discern similarities and differences in rhizome metabolism over the course of a growing season in lowland (Kanlow) and upland (Summer) cultivars of switchgrass with differences in winter survival, with an emphasis on evaluating transcriptional and metabolite changes occurring towards the end of the growing season when plants will be entering dormancy (dormancy onset), and after a killing frost when aerial portions of the plant would die or have fully senesced (dormancy). Kanlow and Summer, and hybrids between these two cultivars, continue to provide genetics for trait improvements [13,14,15].

## 2. Results

### 2.1. ABA Levels and ABA Influenced Genes Increased in Tandem with Progression to Dormancy

**Figure 1:**
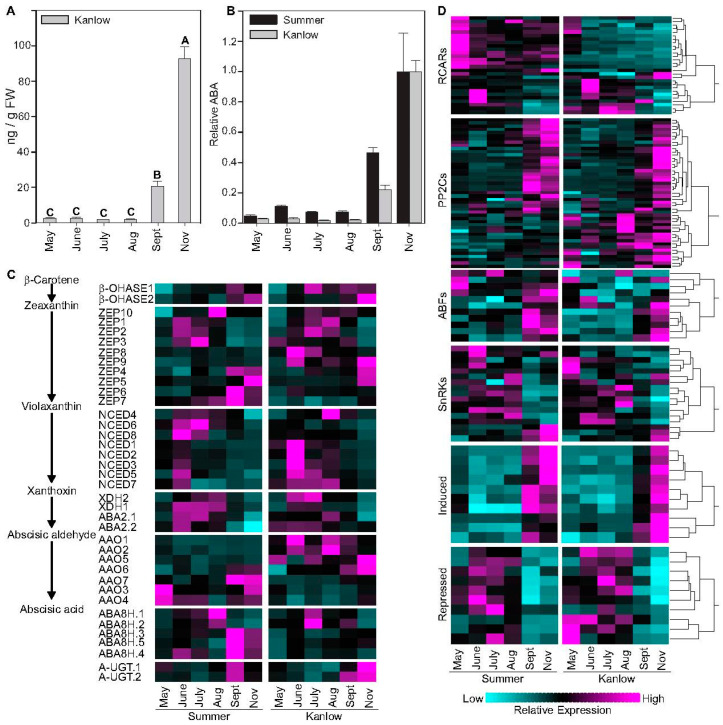
Abscisic acid (ABA) levels and changes in expression patterns of ABA-related genes. (**A**) ABA content in Kanlow rhizomes at each sampling date. Letters over each bar denote statistical significance. Error bars represent standard error. (**B**) Relative ABA levels in Summer (black bars) and Kanlow (grey bars) at each sampling date. ABA data for Summer rhizomes have been previously published [11]. Error bars represent standard error. (**C**) Abbreviated ABA pathway (left) and heat map of genes encoding the various ABA biosynthetic enzymes at each sampling date for Summer and Kanlow rhizomes. (**D**) Changes in expression of genes encoding proteins that are associated with ABA at each sampling time. Gene abbreviations as provided in the text. Cyan is low expression and magenta is high expression. Full list of genes is provided in Supplementary Data S1.

ABA levels in Kanlow rhizomes were low and not significantly different for the first four sampling times, from May through August (Figure 1A). Levels increased substantially, approximately 10-fold, between the August and September samplings, and increased almost 5-fold between the September and November samplings (Figure 1A), indicating a preparation towards dormancy. When ABA levels throughout the season were compared to data obtained previously from Summer rhizomes [11], the seasonal progression in ABA accumulation in rhizomes was quite similar, although relative ABA content was higher in rhizomes collected from Summer plants in September (Figure 1B).

Expression levels of differentially expressed genes in Kanlow rhizomes encoding enzymes needed for ABA biosynthesis, ABA receptors (RCAR), select protein phosphatases 2C (PP2C), ABRE-binding factors (ABF), sucrose non-fermenting kinases (SnRKs), and select genes up/down regulated by ABA were then analyzed and compared to expression profiles previously documented in Summer rhizomes (Figure 1C,D).

Peak expression of a beta-carotene-3-hydroxylase (β-OHASE) was significantly upregulated both in Kanlow rhizomes sampled after a killing frost, and in dormant Summer rhizomes (Figure 1C). β-OHASE produces zeaxanthin, which is converted to violaxanthin by Zeaxanthin epoxidases (ZEP, ABA1). Genes encoding ZEPs were variably enriched, with significant enrichment of several ZEPs in Summer rhizomes sampled in September, and three in Kanlow rhizomes in November. Nine-cis-epoxycarotenoid dioxygenase (NCED) converts violaxanthin to xanthoxin. Eight copies of genes encoding NCEDs were maximally expressed in July and August in both cultivars with significant downregulation in November. Genes encoding the next two enzymes needed for ABA biosynthesis, namely ABA DEFICIENT2 (ABA2) and ALDEHYDE OXIDASE2 (AAO), were expressed differentially (Figure 1C), with *ABA2* being somewhat downregulated in November and *AAO* being upregulated in November. ABA levels can be modulated by biosynthetic and catabolic enzymes, and by conversion to storage forms by glycosyl transferases. ABA 8′-hydroxylases convert ABA to phaseic acid. Five copies of ABA 8′-hydroxylases were expressed in switchgrass rhizomes, with divergent expression profiles in Summer rhizomes compared to Kanlow rhizomes (Figure 1C). Three genes encoding ABA 8′-hydroxylases were maximally expressed in September in Summer rhizomes. Only one ABA 8′-hydroxylase, encoded by Pavir.1NG444600, was dominantly expressed (~75% of all transcripts) with peak expression in July and significant downregulation in November, in Kanlow rhizomes. ABA-UDP-glucosyl transferases catalyze the conjugation of ABA to glucose to maintain ABA in an inactive form; peak expression of two copies encoding putative switchgrass ABA-UDP-glucosyl transferases were detected in Kanlow rhizomes sampled in November, and in Summer rhizomes in September (Figure 1C).

The switchgrass genome contains large numbers of genes encoding RCARs, PP2Cs, and ABFs. Expression of many copies of these genes were detected in switchgrass rhizomes (Figure 1D; Supplementary Data S1). RCARs were frequently highly expressed in rhizomes sampled during periods of active plant growth (May-August samplings), although a few were more highly expressed in rhizomes sampled at the end of the growing season (Figure 1D). However, one RCAR, encoded by Pavir.3NG041000, was upregulated in Kanlow rhizomes obtained after a killing frost in November (Figure 1D).

When liganded with ABA, RCARs can form complexes with clade A PP2Cs, releasing sucrose non-fermenting1-related protein kinases 2 (SnRK2s) from inhibition by PP2Cs. Released SnRK2s subsequently activate downstream signaling that leads to ABA-induced changes in cell functions [16]. Differential expression of 131 PP2Cs was documented in Kanlow rhizomes (Supplementary Data S1), of which 47 were most highly expressed in September and November sampling dates, consistent with increased levels of ABA (Figure 1D). These consisted of genes encoding switchgrass orthologs to Arabidopsis PP2Cs that are highly induced in response to ABA, such as HAI1, HAI2, and HA13. Notably, switchgrass orthologs to Arabidopsis AP2C1 (Pavir.9NG715000), WIN2 (Pavir.4KG380800), and AHG1 (Pavir.7KG093600) were also highly expressed in the November samplings. Among these PP2Cs, the one encoded by Pavir.9NG715000 was most closely related to the Arabidopsis CO_2_ sensor, PP2C5 (AT2G40180) [17], and contains a potentially intrinsically disordered domain at its N-terminal domain. However, the actual role of Pavir.9NG715000 in switchgrass remains to be determined.

Eleven and seventeen copies of ABFs and SnRKs, respectively, were differentially expressed in rhizomes. Among the eleven ABF copies with detectable expression in rhizomes, four had their highest expression in Kanlow rhizomes in November (Figure 1D). These were orthologous to Arabidopsis GBF4 (AT1G03970), ABF3 (AT3G56850), and ABF4 (AT3G19290), indicating a strong responsiveness to increased ABA levels detected in post-frost rhizome samplings. Of the 17 SnRK copies detected, expression of two SnRKs, encoded by Pavir.9NG459300 and Pavir.9KG401100, were induced in rhizomes sampled in November (Figure 1D). Several of the other copies of genes encoding SnRKs were maximally expressed at earlier time points.

In response to the increased ABA content, many genes are induced or repressed in the model plant Arabidopsis [18]. Expression of 68 and 69 switchgrass orthologs of Arabidopsis genes that are induced or repressed by ABA, respectively, were found to be in rhizomes across all sampling dates (Supplementary Data S1). Ten of the most highly induced and most highly repressed genes are shown in Figure 1D. Notably, several of the genes were highly expressed by the September sampling date in Summer rhizomes. Among the most highly induced genes in Kanlow rhizomes (greatest expression in November samplings; see Supplementary Data S1) were a hydroxyproline-rich stress-induced protein, two copies of genes encoding orthologs to Arabidopsis VIRB2-interacting proteins, which regulate intracellular trafficking, a late embryogenesis abundant protein, a β-amylase 1, a β-vacuolar processing enzyme, an AMP-dependent synthetase and ligase family protein, and a zinc finger C-x8-C-x5-C-x3-H type family protein orthologous to ATOFZ2, which confers oxidative stress tolerance and responds to ABA. Arabidopsis orthologs to AB1 five binding protein (AT1G13740), needed for transcription of ABA-induced genes, and a pseudo-response regulator (AT5G24470) that impacts circadian rhythms, were both highly induced in Kanlow and Summer rhizomes.

Similarly, among the ten most highly repressed genes in Kanlow rhizomes collected post-frost, there were some genes whose Arabidopsis orthologs respond to ABA (CRK29, MAX1) and others involved with disease resistance (HRT, AZL1, RPM1), cuticular wax synthesis (CER8), phosphate starvation response (ATPS3), cell wall elongation (BOR2), and two copies of pyruvate-Pi-dikinase (Figure 1D).

### 2.2. Sucrose and Raffinose-Family Oligosaccharides Levels Tracked with Transition to Dormancy

Previously, it had been reported that sucrose content increased in Summer rhizomes at dormancy to support rhizome metabolism [11]. To confirm these findings, select sugars and raffinose-family oligosaccharides (RFOs) were determined in rhizome extracts from both Summer and Kanlow plants at all sampling dates using HPAEC-PAD. Additionally, gene expression of proteins linked to RFOs induction and biosynthesis were identified to establish a possible link between sugar levels and gene expression.

**Figure 2:**
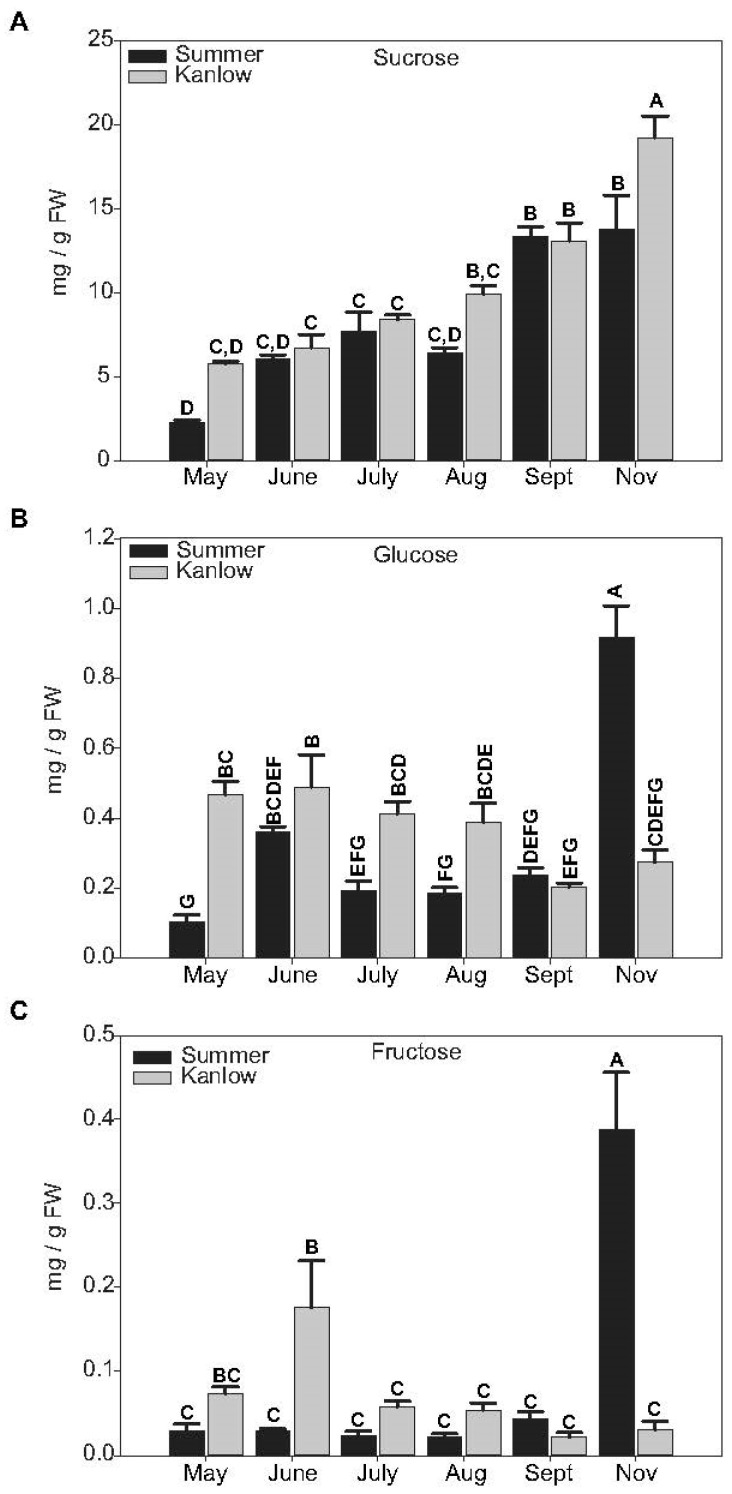
Sugar levels in rhizomes at each sampling date. (**A**) Sucrose. (**B**) Glucose. (**C**) Fructose. In all panels, black bars indicate Summer, and grey bars indicate Kanlow rhizomes. Letters over each bar denote statistical significance. Error bars represent standard error.

Sucrose content (mg g^−1^ FW) increased at each sampling date in both cultivars and was highest in rhizomes collected post killing frost in November (Figure 2A). In November, sucrose levels were significantly greater in Kanlow rhizomes relative to Summer rhizomes. Glucose (Figure 2B) and fructose (Figure 2C) levels were more variable. Glucose content was usually greater in Kanlow rhizomes compared to Summer rhizomes, except in rhizomes collected post frost, when free glucose levels were about 3-fold higher in Summer rhizomes (Figure 2B). Fructose levels were lower than glucose levels across most sampling dates except in June and November, where glucose levels were significantly greater in Kanlow (June) and fructose levels were significantly greater in Summer (November) (Figure 2C).

**Figure 3:**
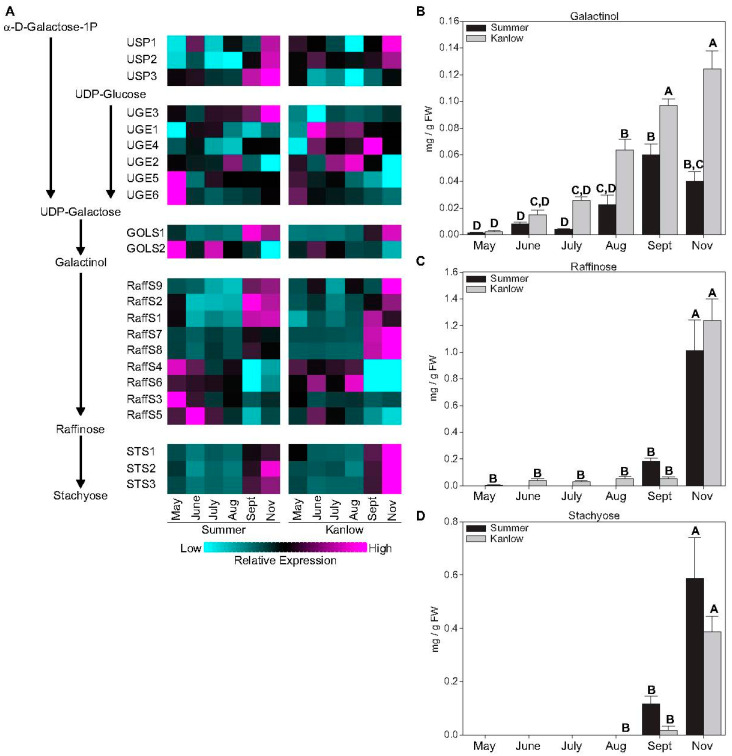
Genes associated with raffinose-family oligosaccharides (RFO) biosynthesis and RFO levels in rhizomes. (**A**) Abbreviated biosynthetic pathway for RFO biosynthesis and heat map of changes in expression of genes encoding the respective enzymes catalyzing each step in RFO biosynthesis. (**B**) Galactinol. (**C**) Raffinose. (**D**) Stachyose. In all panels, black bars indicate Summer, and grey bars indicate Kanlow rhizomes. Letters over each bar denote statistical significance. Error bars represent standard error. For heatmaps, cyan is low expression and magenta is high expression. Full list of genes is provided in Supplementary Data S1.

Several copies of genes associated with RFO biosynthesis were differentially expressed in switchgrass rhizomes (Figure 3A). USP encodes an UTP-sugar pyrophosphorylase, which can form UDP-glucose. Three copies of USP were expressed, and all three were upregulated in rhizomes of both cultivars collected post frost. UDP-glucose is converted to UDP-galactose via the enzyme UDP-glucose/UDP galactose epimerase (UGE; Figure 3A). Of the six copies of UGE, only one, UGE3, was upregulated at later sampling dates, whereas two others, UGE5 and UGE6, were significantly upregulated at green-up. UDP-galactose is the substrate for the enzyme galactinol synthase (GOLS) and produces the first dedicated substrate during RFO biosynthesis. Two copies of GOLS were expressed with differing patterns, one labeled GOLS1 was significantly upregulated in Summer rhizomes at the September sampling date and in November in Kanlow rhizomes. The other copy of GOLS (GOLS2) was upregulated during the early parts of the growing season in both cultivars (Figure 3A). Galactinol produced by GOLS is conjugated successively with sucrose to form raffinose and stachyose. Several raffinose synthases (RaffS; Figure 3A) and three copies of stachyose synthases were strongly upregulated in the September and November harvests in Summer and Kanlow, respectively, (Figure 3A).

The measured levels of RFOs essentially followed the expression levels of genes encoding RFO biosynthetic enzymes (Figure 3A). Galactinol content was consistently higher in Kanlow rhizomes, and significantly greater than galactinol contents in Summer rhizomes in the August to November sampling dates (Figure 3B). Whereas galactinol levels started to increase in August, levels of raffinose (Figure 3C) and stachyose (Figure 3D) were most abundant in rhizomes harvested post-killing frost and frequently below detection levels at the earlier time points. In general, galactinol levels were approximately 10-fold lower (mg g FW^−1^) than the other RFOs across all sampling dates in both cultivars.

### 2.3. Metabolite Levels Differentiate Summer and Kanlow Rhizomes

**Figure 4:**
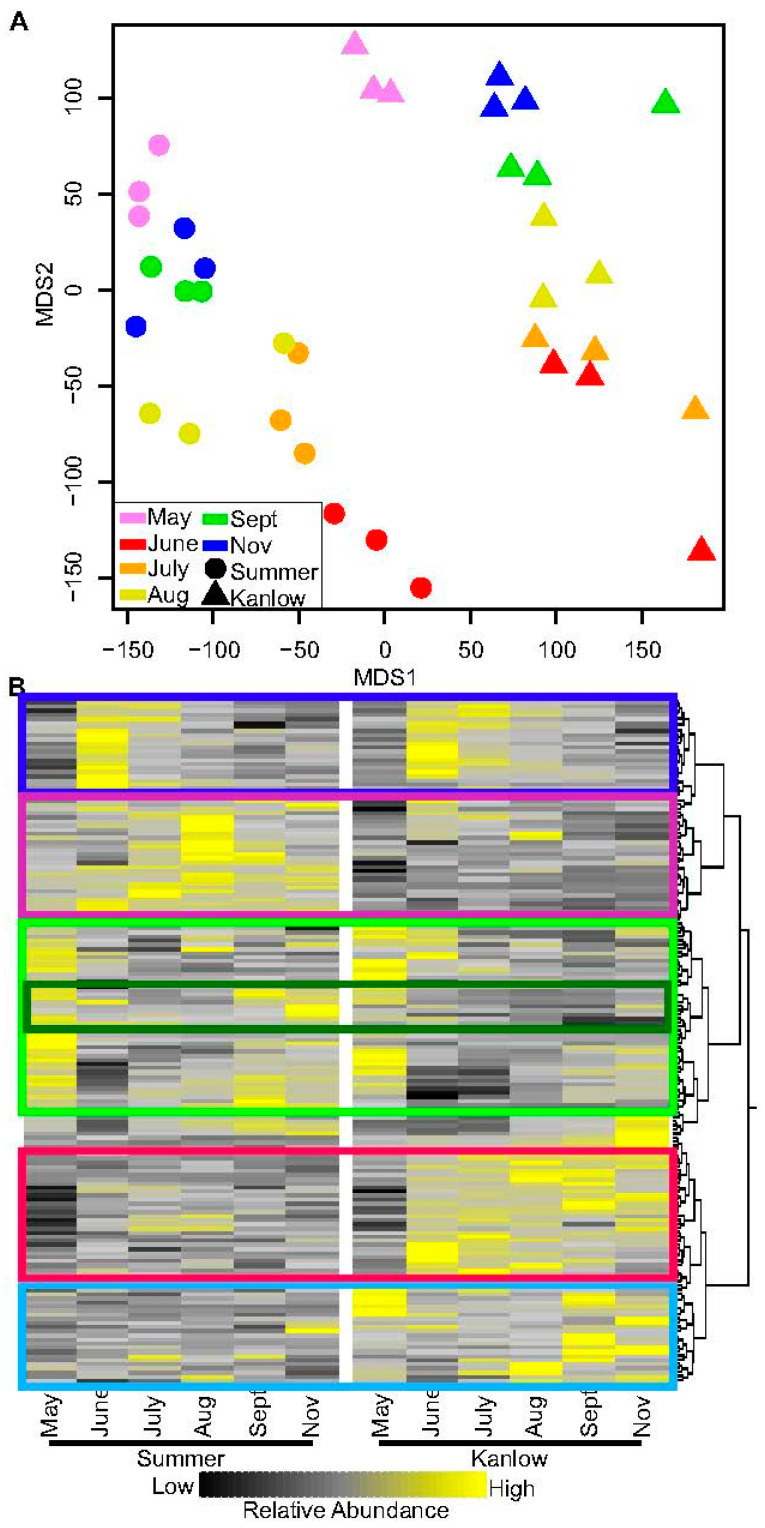
Metabolites present in Kanlow and Summer rhizomes. (**A**) MDS plot of metabolites detected in individual biological replicates obtained from Kanlow (▲) and Summer (●) at each sampling date. May (purple); June (red); July (orange); August (yellow); September (green); and November (blue). (**B**) Individual metabolite heatmap. Boxes indicate metabolites frequently found in greatest abundances in May (green box); June (dark blue box); August (purple box); September (red box); and November (light blue box). Individual metabolite identity is given in Supplementary Data S1 in the order shown in Figure 2B. Black is low abundance and yellow is high abundance.

Metabolite levels in rhizome extracts were quantitated using LCMS (see methods). A total of 219 metabolites were identified, with 166 metabolites being differentially abundant across all harvest dates. Multidimensional scaling (MDS) plots indicated a clear differentiation between Summer and Kanlow rhizomes in MDS1, while timepoints were segregated along MDS2 and were oriented in roughly the same pattern in the two cultivars (Figure 4A), indicating common and unique aspects of seasonal rhizome metabolism in the upland Summer and lowland Kanlow cultivars.

A heatmap of relative metabolite abundances is shown in Figure 4B, where yellow represents higher relative abundance and black represents lower abundances for each metabolite across sampling times and between the two cultivars. Metabolite identities are given in order starting from the top to the bottom of Figure S4 in Supplementary Data S1. There was a seasonal change in metabolite abundances in the rhizomes of both cultivars with enrichment of both common and unique metabolites at each sampling date. For example, at green-up, both Summer and Kanlow rhizomes were enriched in amino acids such as valine, leucine, and phenylalanine, and several dicarboxylic acids such as isocitrate, aconitate, and citraconic acid. They were depleted in levels of alanine, glutamate, lactate, and fumarate (Figure 4B, green box; Supplementary Data S1). Similarly, many metabolites in common between the two cultivars were enriched in rhizomes sampled in June, a period of strong vegetative growth. These included several amino acids, organic acids, and other small molecules required for vitamin and polymer biosynthesis, suggestive of increased growth-related metabolic activities (Figure 4B, blue box; Supplementary Data S1). Notably, other metabolites such as sn-glycerol-3-phosphate, UDP-D-gluconate, and cholesteryl sulfate were more enriched in Kanlow rhizomes relative to Summer rhizomes sampled in June, and remained enriched in Kanlow rhizomes at all later sampling dates (Figure 4B, red box; Supplementary Data S1). A differentiation in the enrichment of metabolite abundances between two cultivars was more pronounced in the July to November sampling times (Figure 4B). Several metabolites, such as pantothenate, which is generated during amino acid metabolism and is needed for CoA biosynthesis; pyrroline-5-carboxylate, an intermediate in proline biosynthesis; nicotinamide, a precursor of vitamin B3 and needed for NAD biosynthesis; and S-adenosyl-L-methionine, needed for methylation and ethylene biosynthesis, were enriched in Summer rhizomes sampled in August, and many of these metabolites decreased in relative abundances in at the next two sampling times (Figure 4B, purple box; Supplementary Data S1).

Metabolites with higher relative enrichment in Kanlow rhizomes sampled in July included betaine aldehyde, an intermediate in glycine metabolism and a precursor for the osmoprotectant betaine; fructose-1,6-bisphosphate; sn-glycerol-3-phosphate; ribose phosphate; and 2-deoxyglucose-6-phosphate. Many of these metabolites remained at higher relative levels in Kanlow rhizomes at later sampling dates as well (Figure 4B).

End-of-season sampling in November also suggested subtle differences in rhizome metabolism in the upland versus lowland cultivars (Figure 4B). Several products arising from catabolism of amino acids and nucleic acids, such as purine, cystathionine, phenyllactic acid, and acetyl lysine were more enriched in Summer rhizomes as compared to Kanlow rhizomes. In contrast, thiamine, adenine, oxaloacetate, allantoate, and geranyl pyrophosphate were more enriched in Kanlow rhizomes.

### 2.4. Kanlow and Summer Transcriptomes Were Differentiated over the Growing Season

**Figure 5:**
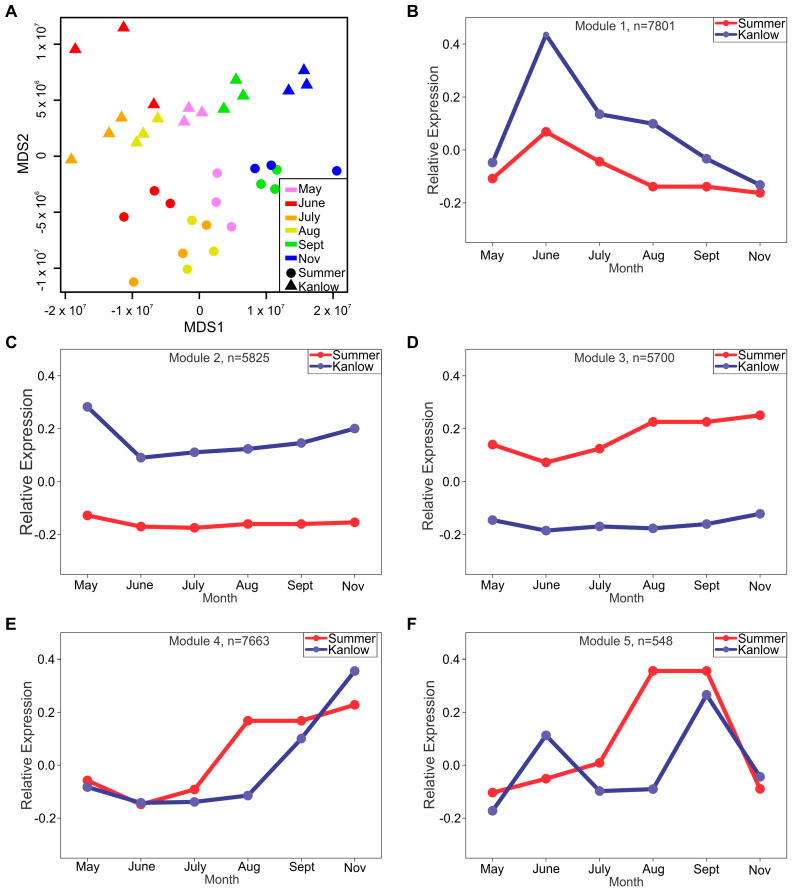
Global gene expression analysis in switchgrass rhizomes. (**A**) MDS plot of transcriptomes for every biological replicate at each sampling date for Kanlow (▲) and Summer (●) rhizomes. Colors are as described in Figure 4A. (**B**) Network analysis of transcriptomic data. (**C**–**F**) Expression profiles for modules (1–5) described in the text are shown. Kanlow (blue lines) and Summer (red lines) at each sampling date. Full results from network analyses are given in Supplementary Data S1.

An MDS plot of transcriptomes over the course of the growing season is shown in Figure 5A. Summer and Kanlow transcriptomes were primarily differentiated on the MDS2 axis, with differentiation at each sampling date within and between the two cultivars occurring from the May collection date to the post-frost sampling in November along MDS1 (Figure 5A). Interestingly, the May timepoints were found in the middle of the plot, separating the “growth” samples collected in June, July, and August from the “dormancy” samples collected in September and November.

Network analyses of gene expression resulted in the identification of 11 coexpression modules (Supplementary Data S1). Five select modules with the expression patterns that correlate most strongly with anticipated metabolic changes that occurred in rhizomes over the growing season are shown in Figure 5B–F to highlight the similarities and differences in expression profiles in Summer and Kanlow rhizomes. Gene ontology (GO) enrichment was performed on the five chosen modules to obtain processes and functions that were significantly enriched within each module. Module M1 is comprised of 7901 genes that had the highest expression in June in both cultivars, although relative expression was much greater in Kanlow (Figure 5B). This module was significantly enriched with 45 gene ontology-biological processes (GO:BP) and 54 GO:molecular functions (GO:MF) terms at *p* ≤ 0.05 (Supplementary Data S1). Many of these GO:BP and GO:MF terms indicated active cell metabolic processes linked to growth. The GO:BP terms included among others, response to oxidative stress, carbohydrate metabolic process, oxidation-reduction process, photosynthesis, and cell wall modification cellulose biosynthetic process. The GO:MF terms enriched included peroxidase activity, protein kinase activity, oxidoreductase activity acting on NAD(P), proton transporting ATP synthase activity, and glutamate-ammonia ligase activity, among others (Supplementary Data S1). M1 enrichment for KEGG pathways found 14 pathways that were significantly enriched (Supplementary Data S1), largely consistent with GO analyses. At the gene level, there were significant numbers encoding ribosomal proteins, proteins required for photosynthesis, and plastid functions, likely arising from tiller initials present on the rhizomes, and other cellular biosynthetic activities that were consistent with active plant growth.

Modules M2 and M3 consisted of genes that were more highly expressed in Kanlow (M2) or Summer (M3) rhizomes at all sampling dates (Figure 5B,C), with peak expression occurring in May and gradually increasing towards the end of the season. M2 was comprised of 5825 genes and enriched with 28 GO:BP and 35 GO:MF terms, whereas M3 was comprised of 5700 genes and enriched with 83 GO:BP terms and 65 GO:MF terms (Supplementary Data S1). Significantly enriched M2 GO:BP terms indicated a dominance of growth and transport-related processes. These included the purine nucleotide biosynthetic process, nitrogen compound metabolic process, intercellular transport, ribosome biogenesis, protein methylation, and chromatin remodeling. In turn, the significantly enriched GO:MF terms included nucleic acid binding (which was also enriched in M3), DNA-directed DNA polymerase activity, polysaccharide binding, and several hydrolase terms. No significant KEGG enrichment was found in M2 (Supplementary Data S1).

GO terms enrichment was greater at both the GO:BP and GO:MF levels in M3 as compared to M2 (Figure 5C), and included a number of terms associated with polymer assembly and processing, such as protein folding, RNA processing, iron-sulfur cluster assembly, DNA repair, and phospholipid biosynthetic process. Other terms indicated cellular events linked to both biosynthesis and degradation of polymers and metabolites; for example, sucrose metabolic process, nucleotide biosynthetic process, versus, autophagy, and proteolysis. In contrast to M2, three KEGG pathways (diterpenoid biosynthesis, tryptophan metabolism and glutathione metabolism) were significantly enriched in M3 (Supplementary Data S1).

M4 contained 7663 genes that had strong end-of-the-year expression profiles in both cultivars and are likely linked to processes common during switchgrasses’ transition to dormancy. Notably, expression of these genes significantly increased in August and remained high through November in Summer rhizomes but did not begin to increase in expression in Kanlow until September and reached their peak expression in November (Figure 5E). M4 was enriched in 12 GO:BP terms and 21 GO:MF terms. The GO:BP terms included protein phosphorylation (also enriched in M5), regulation of translational elongation, anion transport, and microtubule cytoskeleton organization. Notably, carbon fixation and the TCA cycle were part of the enriched terms. In concordance with the GO:BP enrichment, GO:MF included ADP and ATP binding, protein kinase activity, and calcium-dependent phosphorylation. Similarly, phosphoenolpyruvate carboxylase and enzyme regulator activity were enriched. KEGG enrichment analysis indicated that five pathways (biotin metabolism, spliceosome, homologous recombination, nicotinate and nicotinamide metabolism, and proteosome) were significantly enriched in M4 (Supplementary Data S1).

M5 (Figure 5F) contained a smaller cluster of 548 genes with some similarities and differences in expression patterns in Kanlow versus Summer rhizomes. These genes had bimodal expression in Kanlow rhizomes, with peak expression at the June and September sampling dates. These same genes did not have a high expression at the June sampling date in Summer but were highly expressed at the August and September sampling dates. The eight GO:BP terms enriched in this module were associated with cell division and cell growth, and included cytokinin metabolic process, regulation of transcription, protein phosphorylation, and regulation of mitotic metaphase/anaphase. There were 12 significantly enriched GO:MF terms in M10 (Supplementary Data S1), which included two transporter activities, transferase activity, protein kinase activity, and cytokinin dehydrogenase activity. At the gene level, this module had several genes encoding proteins needed for ribosomal assembly and protein synthesis. Consistent with gene enrichment in this module, two KEGG pathways, namely ribosome and ribosome biogenesis in eukaryotes, were significantly enriched (Supplementary Data S1).

An analysis of Pearson correlations for metabolite abundances correlated with each module eigengenes identified by network analysis was performed (Supplementary Data S1). These correlations identify the interrelationships between gene expression and metabolite levels across all sampling dates, and therefore can be positive or negative. A positive correlation indicates that metabolite levels tracked in a similar manner with the gene expression profile of the module, while a negative correlation indicates that metabolite levels tracked in manner opposite to gene expression profiles for a specific module. M1 was significantly correlated both positively with 47 and negatively with 32 metabolites, including most of the amino acids, sugars, nucleic acids, organic acids, and their derivatives. Thirteen of these significantly correlated metabolites are also found in six KEGG pathways enriched in M1 (Supplementary Data S1). M2 was positively correlated with 42 metabolites and negatively correlated with 38 metabolites. Positively correlated metabolites included serine, p-aminobenzoate, and UDP-D-glucuronate, to name a few. Negatively correlated metabolites included sedoheptulose-1-7-bisphosphate, glucono-D-lactone, and nicotinate (Supplementary Data S1). Many of the metabolites significantly positively correlated to M2 gene expression profiles were significantly negatively correlated to M3 gene expression profiles, and included adenine, folate, and UDP-glucuronate. Conversely, sedoheptulose-1-7-bisphosphate, glucono-D-lactone, and nicotinate were positively correlated with M3. Thirty-one metabolites were negatively correlated to gene expression profiles in M4 and included pipecolic acid and a number of carboxylic acids. Twenty-three metabolites were significantly positively correlated to gene expression in M4, and included several sugar phosphates, citrate, and some amino acids, such as proline and arginine. Six of these significantly correlated metabolites are also found in two KEGG pathways enriched in M4 (Supplementary Data S1). For M5, there were nine and four metabolites that were positively and negatively correlated, respectively (Supplementary Data S1).

### 2.5. Transporter Gene Families Were More Abundant in Specific Modules

To discern potential differences and similarities in the expression of transporter encoding genes, their abundances in individual modules were performed (Supplementary Data S1). Transporters present in M1–M5 are given in Table 1.

M1 contained a total of 117 genes encoding switchgrass transporters, of which 32 and 22 were annotated as peptide transporters (PTR) and major facilitator superfamily proteins (MFSP), respectively. M4 had fewer PTRs (6) and greater numbers of MFSP (31), Na/H^+^ exchangers (8), and calcium ATPase 2 (ACA; 11). M5 only contained two transporters, one divalent anion/Na^+^ symporter (LSI), and one phosphate transporter (PHT). A comparison between the M2 (Kanlow) and M3 (Summer) modules indicated almost a two-fold increase in the number of annotated transporter genes in the Summer module (M3; 103; Supplementary Data S1) relative to the Kanlow module (M2; 59; Supplementary Data S1). Select classes are shown in Table 1. The numbers of ACA, cation-efflux exchangers (CAX), high affinity potassium transporters (KUP), MFSP, Na/H^+^ exchangers, PHT, and CAX were greater in M3 versus M2. Notably, two vacuolar iron transporter (VIT) and one LSI were found only in M3.

### 2.6. Transcription Factors Were Differentially Enriched in Modules 1–5

A total of 56 classes of genes encoding switchgrass TFs were detected within all the modules (Supplementary Data S1). Select TFs present in M1–M5 are given in Table 2. M1 had the highest numbers of TFs (447; Supplementary Data S1) and had the highest abundance of several classes of TFs relative to the other modules. Particularly striking were the high numbers of bHLH, bZIP, ERF, NAC, MYB, WRKY, YABBY, and HD-Zip families of TFs. Many of these TFs have been implicated in growth processes in model plants. Gene expression in M1 was greatest in June, when plant growth was accelerating.

M4 contained 271 genes annotated as TFs (Supplementary Data S1). These included 5 members of the basic pentacysteine 1 (BBR-BPC) found only in M4. Members of the BBR-BPC regulate a number of growth and developmental processes in plants [19]. Other genes encoding TFs with greater abundances in M4 included 23 CCCH-type zinc finger proteins (C3H), eight TESMIN/TSO1-like CXC 2 (CPP), five GL1 enhancer binding proteins (GeBP), seven homeodomain-like transcriptional regulators (HB-other), 12 heat shock factors (HSF), and 25 MYB-related.

M2 and M3 contained a total of 173 and 221 TFs, respectively (Supplementary Data S1). Although members of the major classes of plant TFs such as WRKY, bHLH, ERF, and NAC were represented in approximately similar numbers, genes encoding other TF classes were uniquely abundant in the Kanlow (M2) or Summer (M3). M3 also contained more HSF and MYB genes as compared to M2 (Table 2; Supplementary Data S1). M5 contained a total of eight TF encoding genes that included one HSF, one LBD, and two MYBs (Table 2; Supplementary Data S1).

## 3. Discussion

Switchgrass occurs as two ecotypes, upland and lowland [4]. The upland ecotypes are adapted to more xeric sites in the northern latitudes, while the lowland ecotypes are adapted to wetter, more southern latitudes. Aside from their differential zones of adaptation, lowland switchgrass plants yield higher biomass as compared to the upland ecotypes, and lowland plants frequently suffer from winter kill in northern latitudes [12]—although there is an extended range of winter survival among lowland plants [7,20].

The physiological basis for the differences in winter adaptations are still unclear, although a recent detailed study of cold acclimation among diverse switchgrass accessions has been performed [21]. These authors found that the more northern lines develop cold acclimation at a higher threshold temperature compared to the more southern lines, with photoperiod playing a role in these processes. Overall, their results indicated that better responses to cold and freezing occurred sooner in the northern lines relative to the southern lines, permitting greater winter survival [21].

ABA has important and diverse roles in controlling plant physiological processes, including dormancy [22,23,24]. ABA binding to receptors in cells acts as a trigger that modulates gene expression and brings about changes in cellular metabolism. Many of these genes and cellular responses influenced by ABA and its receptors are well conserved across plant species, and provide a means to correlate changes in ABA levels to changes in gene expression and levels of specific metabolites. ABA was implicated in the transition to dormancy in switchgrass rhizomes sampled from Summer plants [11]. Likewise, in the current study, a role for ABA during dormancy onset in Kanlow rhizomes was established. However, the relative levels of ABA in Kanlow rhizomes were significantly different from Summer rhizomes only in the September harvests, pointing to subtle differences in the timing for dormancy onset between the two cultivars. Earlier work had indicated that aerial senescence was at an advanced stage in Summer plants relative to Kanlow plants during this sampling time [25]. These results suggest that aerial senescence impacts the onset of rhizome dormancy, potentially by intersecting with ABA transport, biosynthesis, and/or conversion of inactive storage forms of ABA to active hormones in rhizomes. Notably, the expression of several genes in Kanlow rhizomes encoding ABA biosynthetic enzymes, such as zeaxanthin epoxidase (ABA1), nine-cis-epoxycarotenoid dioxygenase (NCED), and ABA2, occurred during the growth phases of the plant (June–August). It is plausible that roots could be a source of ABA transported to rhizomes during the onset of dormancy. Although the origin of increased ABA levels in rhizomes remains somewhat unclear, there was abundant evidence for ABA-dependent changes in transcription and metabolism in Kanlow rhizomes, reinforcing the importance of ABA to remodeling rhizome metabolism in switchgrass dormancy.

Sucrose levels increased to a maximum with the transition to dormancy in Kanlow rhizomes, as has been reported for Summer rhizomes [11], although free sucrose levels were significantly greater in Kanlow rhizomes in November compared to Summer rhizomes. In contrast, free glucose and fructose levels were significantly elevated in dormant Summer rhizomes, indicating differences in dormant rhizome metabolism. Summer rhizomes are elongated, intertwining rhizomatous structures, whereas Kanlow rhizomes are caespitose, with short rhizomes [4]. It is unclear if rhizome morphology influenced sugar levels. Alternatively, differences in starch metabolism might have impacted free hexose pools. Published data [11,25] suggest that Summer rhizomes were in a more advanced state of dormancy in the November sampling time relative to Kanlow. Since starch is the major source of stored energy during dormancy, Summer rhizomes could be metabolizing starch to a greater extent than Kanlow rhizomes, which would have more recently transitioned to a dormant state.

Based on transcriptomic data, there was a clear association in the activation of RFO biosynthesis in Summer rhizomes at dormancy, which appeared to be impacted by an HSF TF [11]. To extend these findings, actual RFO contents were measured in Summer and Kanlow rhizomes. Measured RFO levels and the expression of genes encoding enzymes in this pathway were enhanced in both Kanlow and Summer rhizomes collected in November, indicating a role for these sugars in switchgrass rhizome dormancy. Since RFOs can serve many purposes in plant cells [26,27], it is likely that these oligosaccharides serve roles in osmotic protection and oxidative stress during dormancy.

Other metabolites such as amino acids, sugars, and organic acids displayed both a seasonal as well as cultivar-specific accumulation; most notably, there were greater similarities during the growth phases, especially at the June sampling date, with somewhat greater divergence at later sampling times. These changes could be a mixture of cultivar-specific metabolism [25,28,29,30,31], and coupled to the delay in dormancy onset in the lowland versus upland cultivars [21]. Network analyses indicated similar patterns of gene expression in both cultivars in June and towards the end of the year. In both M1 and M4, there was an enhanced representation of genes encoding proteins linked to growth in June (M1), and those associated with ABA and dormancy in November (M4). Expression patterns in M4 indicated that dormancy-related changes in the northerly adapted Summer cultivar began to occur almost a full month before they occurred in the southerly adapted Kanlow, in agreement with data from Willick and Lowry [21]. However, one module (M5) displayed a bimodal expression pattern in Kanlow rhizomes, and a unimodal expression in Summer rhizomes. Notably, this module was enriched in genes encoding ribosomal proteins, suggesting that it may be associated with the remodeling of protein biosynthesis needed to accommodate key developmental events such as the transition to dormancy.

Network analysis identified modules with higher relative gene expression in either Kanlow or Summer rhizomes. These modules were also differentially enriched with TFs, metabolites, GO terms, and KEGG pathways. However, neither module was specifically associated with a developmental transition (for example growth or dormancy), suggesting a link to cultivar-specific events, and possibly to other determining factors such as rhizome type, root, and tiller initiation.

Overall, these data support a delay in the dormancy transition in the lowland cultivar Kanlow relative to the upland cultivar Summer as suggested earlier [12,21]. Recently, genomic selection was used to identify interactions between flowering (heading dates) and winter survival in diverse lowland switchgrass populations [32]. Although positive predictive ability was determined for both traits, the mortality of plants affected by winter successfully identified cold-tolerant genotypes. In this regard, it will be effective to identify these surviving genotypes and conduct detailed biochemical analyses to determine what may be involved in driving winter survival at the cellular level. Given the polyploidy of switchgrass, small but consistent differences in gene expression and protein turnover could be adequate to enhance winter survival in cold-intolerant genotypes.

## 4. Materials and Methods

### 4.1. Plant Growth and Sampling

Kanlow and Summer plants were raised from seeds in a greenhouse, and hand transplanted at sward densities into a field site near Mead, Nebraska, USA. Rhizomes were sampled from three individual plants (genotypes) at five time-points that approximately corresponded to spring green-up (May), rapid vegetative growth (June), transition to flowering (July), early flowering (August), seed set (September), and after a killing frost (November). Other environmental details at this field site have been provided earlier (Palmer et al., 2017). Sampling dates and environmental conditions at this field site have been described earlier [11]. Sampled rhizomes were cleaned in the field, flash-frozen with liquid N_2_, and stored at −80 °C until analyzed. Prior to analysis, rhizomes were cryogenically ground using a SPEX 6850 Freezer Mill instrument. Aliquots of ground tissues were used for subsequent downstream analyses.

### 4.2. Transcriptomics

RNA extraction and subsequent RNA-Seq analysis was performed as described earlier [11]. Briefly, 100 bp SE sequencing of mRNA isolated from all 18 samples (6 sampling dates × 3 biological replicates) was carried out using an Illumina HiSeq2500 system. An average of 55 M quality reads were generated for each sample. Reads were mapped to version 4.1 of the switchgrass genome (phytozome.jgi.doe.gov) using HISAT2 [33] and reads were assigned to gene features using featureCounts [34]. An average of 88% of the reads mapped uniquely to the genome, with an average of 68% of the reads mapping to the annotated exonic gene space. Differential gene expression was calculated using DESeq2 [35] with differentially expressed genes (DEGs) requiring an adjusted *p*-value < 0.05 and a |log_2_ fold change| > 1.0. Co-expression analysis was done using the Weighted Gene Co-expression Network Analysis (WGCNA) package in R [36] to create a signed network using power = 16, minModuleSize = 30, and mergeCutHeight = 0.4 parameters.

### 4.3. Metabolite Analyses

Triplicate 50 ± 2 mg aliquots of each sample were analyzed for ABA content using mass spectrometry, essentially as described earlier [37,38].

Polar metabolites were extracted from 50 ± 2 mg of ground tissue in 80% methanol and analyzed by multiple reaction monitoring (LC-MRM-MS) analysis as described by Koch et al. [39]. Metabolite data were analyzed using the MetaboAnalyst [40] workflow. Abundance values were log transformed and differentially abundant metabolites were identified using the ANOVA2 test.

### 4.4. Water Soluble Carbohydrates (WSC)

Water soluble sugars and galactinol were analyzed with high-performance anion-exchange chromatography-pulsed amperometric detection (HPAEC-PAD). Analysis of the major, extractable soluble oligosaccharide products was performed by HPAEC-PAD, due to its superior sensitivity, speed, and separation chemistry for soluble oligosaccharide components [41]. To obtain the WSCs, approximately 100 ± 2 mg (fresh weight) of cryogenically ground rhizome samples were extracted with 1000 µL of 80% ethanol:20% 18 MΩ water, containing 400 µg mL^−1^ melibiose internal standard for 90 min at 25 °C. After incubation, samples were centrifuged at 13,000× *g* for 10 min and multiple 50 µL aliquots of supernatant from each sample were dried *in vacuo*. To determine soluble sugars, two separate analyses were performed. Under the HPAEC-PAD chromatographic conditions used, galactinol eluted the earliest of the analytes of interest. Due to background signals in the injection void and the lower quantities of galactinol in the samples, quantification was not reliable. Therefore, a solid-phase extraction (SPE) was performed using monolithic aminopropyl-functionalized silica to remove background contaminants and concentrate samples for galactinol measurements.

For WSC determination, 1000 µL of 18 MΩ water was added to one 50 µL aliquot of each sample for soluble carbohydrate analysis of the major soluble sugars (glucose, fructose, sucrose, raffinose, stachyose, verbascose). These extractable soluble sugars and oligosaccharides were determined quantitatively using HPAEC-PAD (Thermo Scientific ICS 5000, Waltham, MA, USA). Samples were maintained at 4 °C prior to analysis and 5 µL of the reconstituted sample was injected onto a Thermo Scientific PA-10 column (2 mm × 250 mm column) at 0.25 mL/min, running 90 mM NaOH isocratically for 30 min [42]. The procedure was performed in triplicate for each sample (3 biological replicates × 3 technical replicates). Analytes were identified and quantified based on a 4-point standard curve generated from authentic standards run several times over the course of analysis.

### 4.5. Galactinol Analysis

Separate 50 µL aliquots were resuspended in 50 µL water, followed by the addition of 450 µL acetonitrile. Each sample was added to a preconditioned (50% acetonitrile:water:0.1% formic acid, then 90% acetonitrile:water:0.1% formic acid) MonoSpin NH_2_ SPE spin column. Samples (500 µL) were centrifuged through the SPE-packing material at 5000 rpm for 30 s, washed with 500 µL 90% acetonitrile:water:0.1% formic acid, then eluted with 500 µL 50% acetonitrile:water:0.1% formic acid. The eluate and the combined loading and wash fractions from each sample were dried *in vacuo*. Samples were resuspended in 250 µL 18 MΩ water, followed by HPAEC-PAD using the conditions describe above. Galactinol was identified and quantified based on a 3-point standard curve generated from an authentic standard run several times over the course of analysis. The combined loading and wash fractions were analyzed to show that no galactinol was present in these fractions. The procedure was performed in triplicate as described for the WSC.

Galactinol, fructose, sucrose, raffinose, stachyose, and melibiose standards were purchased from Sigma-Aldrich Co. (St. Louis, MO, USA). Verbascose was purchased from Megazyme International Ireland Ltd. (Wicklow, Ireland). Glucose, acetonitrile (HPLC grade), and sodium hydroxide (50% *w*/*w*) were purchased from Fisher Scientific. Formic acid (for mass spectrometry) was purchased from Fluka Chemical (Buchs, Switzerland). MonoSpin NH2 SPE spin columns were purchased from GL Sciences, Inc. (Torrance, CA, USA).

### 4.6. Bioinformatic and Statistical Analyses

ANOVA and related statistical analyses were performed in JMP 12.2.0 (SAS Institute, Cary, NC, USA), with mean separation done using Tukey’s HSD test. Gene ontology enrichment of co-expression modules was done using the topGO package in R with the “weight01” algorithm and “fisher” statistic. KEGG pathway enrichment of co-expression modules was done using the GeneOverlap package in R and required a KEGG pathway to have at least five member genes detected in the RNA-Seq data. Significant correlations between metabolites and co-expression module eigengenes were detected using the rcorr function in the Hmisc package in R with the Pearson correlation. Other relevant bioinformatic analyses were as described earlier [11,28,39,43].

## Figures and Tables

**Table 1:** Numbers of select genes encoding different classes of transporters found in Modules 1–5. Full listing of transporters found in this study is given in Supplementary Data S1. Abbreviations are defined in the appropriate section of the text.

Transporter Class	M1	M2	M3	M4	M5
ACA	6	6	15	11	0
CAX	5	1	5	7	0
KUP	9	5	12	5	0
Major Facilitator	22	5	21	31	0
Na/H Exchanger	1	1	5	8	0
PHT	6	1	5	2	1
PTR	32	15	13	6	0
VIT	0	0	2	2	0
LSI	0	0	1	0	1

**Table 2:** Numbers of select genes encoding transcription factors (TFs) found in Modules 1–5. Full listing of all TFs found in this study is given in Supplementary Data S1. TF classes are shown as their commonly abbreviated names.

TF Family	M1	M2	M3	M4	M5
BBR-BPC	0	0	0	5	0
bHLH	53	16	14	9	0
bZIP	32	11	20	25	0
C3H	8	5	9	23	0
CPP	3	2	1	8	0
ERF	40	9	13	5	0
GeBP	1	1	3	5	0
HB	1	1	0	7	0
HD-ZIP	22	7	8	6	0
HSF	4	1	5	12	1
MYB	37	4	14	13	2
MYB-related	13	5	8	25	1
NAC	27	16	22	12	0
WRKY	17	11	13	8	0
YABBY	7	0	0	0	0

## Data Availability

Sequencing data is available under the SRA BioProject accession: PRJNA528958.

## Supplementary Materials

The following supporting information can be downloaded at: https://www.mdpi.com/article/10.3390/plants12081732/s1, Data S1: All gene annotations, expression data, metabolites identified, and full data from network analyses.
